# Still the one: Transcriptional control across leukopoiesis by SPI1/PU.1

**DOI:** 10.70962/jhi.20260152

**Published:** 2026-11-12

**Authors:** Joseph Mackie, Sangwan Sim, Lauren Y. Cominsky, Peter A. Nigrovic, Neil Romberg

**Affiliations:** 1 Division of Immunology and Allergy, Children’s Hospital of Philadelphia, Philadelphia, PA, USA; 2 Division of Immunology, Boston Children’s Hospital, Harvard Medical School, Boston, MA, USA; 3 Immunology Graduate Group, Perelman School of Medicine, University of Pennsylvania, Philadelphia, PA, USA; 4 Division of Rheumatology, Inflammation, and Immunity, Brigham and Women’s Hospital, Harvard Medical School, Boston, MA, USA; 5 Department of Pediatrics, Perelman School of Medicine, Philadelphia, PA, USA; 6 Institute for Immunology and Immune Health, Perelman School of Medicine, University of Pennsylvania, Philadelphia, PA, USA

## Abstract

The transcription factor (TF) SPI1 (historically called PU.1) is a critical pioneer TF and regulator of hematopoiesis. It exerts exquisitely dose-dependent and stage-specific control over lymphoid, myeloid, and central nervous system–resident lineages. Structurally characterized by an intrinsically disordered N-terminal transactivation region and a highly conserved C-terminal DNA-binding E26 transformation–specific domain, SPI1 can operate alone as a monomer, via homodimerization, through epigenetic interactions, and in context-dependent transcriptional partnerships. While early animal models established its essentiality for leukopoiesis, modern human genomics has unveiled direct links between altered SPI1 function and disease. Heterozygous loss-of-function germline variants cause PU.1-mediated agammaglobulinemia, whereas somatic mutations and/or regulatory alterations can drive hematologic malignancies. Dysregulated SPI1 activity is also implicated in autoimmune diseases, including rheumatoid arthritis and systemic lupus erythematosus, and neurodegenerative disorders. This review reconciles foundational murine paradigms with emerging human translational data, highlights current therapeutic landscapes, and projects how single-cell multi-omics and advanced gene editing may shape future directions in the SPI1 biomedical field.

## Teaser

The transcription factor SPI1 (PU.1) regulates leukopoiesis with transcriptional partners in a dose- and stage-specific manner. Disordered SPI1 expression or function is associated with agammaglobulinemia, hematologic malignancies, autoimmune diseases, and neurodegeneration, highlighting its biologic relevance to immunity and immune-mediated disease.

## Introduction

SPI1 is a hematopoietic transcription factor (TF) so named by Moreau-Gachelin and colleagues in 1988 for the activating spleen focus forming virus proviral integration site 1 positioned just upstream of the gene encoding it (1, 2). In mice, virally driven SPI1 expression promotes erythropoiesis at the proerythroblastic stage, resulting in erythroleukemia (1). In independent experiments, SPI1 was identified in 1990 by Klemsz and colleagues from a murine macrophage cDNA library. They named the protein PU.1 for its ability to bind the “PU-box,” a purine-rich nucleic acid sequence recognized by all members of the E26 transformation–specific domain (ETS) TF family (3). Upon inspection of published SPI1 and PU.1 amino acid sequences, they were found to be the same protein (4). In this review, we will follow International Protein Nomenclature Guidelines and refer to the protein as SPI1 and its encoding gene as *SPI1* (human) or *Spi1* (murine).

Nearly 40 years after first recognition of its transformative power, SPI1 remains an active area of scientific exploration due to its exquisitely dose-dependent transcriptional effects, numerous transcriptional partners, multiple modes of regulation, and its stage-specific expression patterns across hematopoietic lineages. Moreover, recent advancements in human genome sequencing have uncovered inborn and somatically acquired *SPI1* variants that pose significant consequences for the health of affected patients. Herein, we will review consensus and controversies in the field of SPI1 biology before shifting to its role in human health and disease states.

## Molecular considerations

### Key features of the SPI1 protein

**Figure 1.**
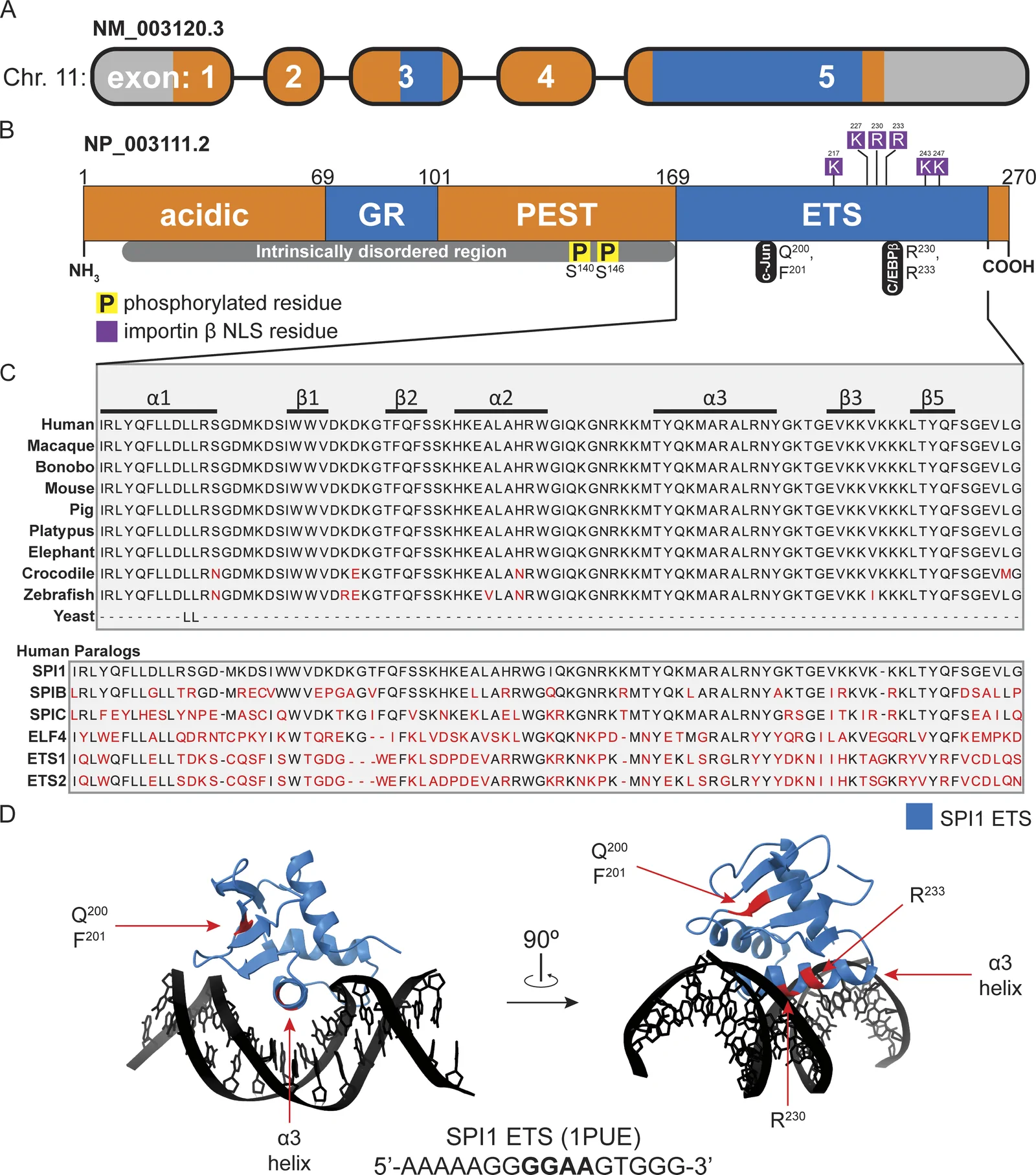
**Molecular features of pioneer TF SPI1. (A)** The human *SPI1* gene encoding a five-exon MANE transcript. Protein-coding functional domains are denoted in orange and blue. **(B)** Map of SPI1 protein functional domains, including acidic, GR, PEST, and the ETS. Key molecular features, including the IDR, phosphoserine sites, residues of heterodimerization, and nuclear localization sequences (NLS), are denoted throughout the structure. **(C)** Evolutionary conservation of SPI1 amino acids in the ETS domain is shown for orthologs (top panel) and human paralogs (bottom panel). Key secondary structures (α helices and β sheets) are denoted throughout. **(D)** Protein Data Bank crystal structure (1PUE) of the SPI1 ETS domain in contact with the 5′-GGAA-3′ SPI1 consensus DNA sequence. Key residues involved in protein–protein and protein–DNA interactions are denoted in red. MANE, matched annotation from NCBI and EBI.

The human *SPI1* gene on chromosome 11 is comprised of five exons and encodes seven isoforms. The most abundant transcript is the matched annotation from National Center for Biotechnology Information (NCBI) and European Bioinformatics Institute (EBI) isoform NM_003120.1. The translated SPI1 protein (NP_003111) is 270 amino acids in length and contains four functional domains: the acidic transactivation, the guanine-rich (GR), the proline, glutamic acid, serine, and threonine-rich (PEST), and the DNA-binding ETS domains (Fig. 1, A and B). There is a high degree of evolutionary conservation between human SPI1 and its orthologs. This is especially true within the ETS domain, where identity to human SPI1 is 100% for most mammals and even monotremes (Fig. 1 C), indicating a sequence under high selective pressure.

One-to-one crystal structures of the SPI1 ETS domain bound to its canonical purine-rich 5′-GGAA-3′ DNA motif reveal a loop-helix-loop structure, a hallmark of ETS-family TFs (5). Conserved arginine residues (R^230^ and R^233^) within the SPI1 α three helix (α3) form hydrogen bonds with the major groove of DNA. The complex is further stabilized by two lysine contacts with the DNA minor groove, K^217^ between the α2 and α3 helices and K^243^ in the wing between the β3 and β5 sheets (Fig. 1, C and D) (5, 6). SPI1 DNA specificity has been exhaustively investigated through foundational chromatin immunoprecipitation sequencing (ChIP-seq) studies, which depict a core SPI1 motif (5′-GGAA-3′) that is largely agnostic to cell type (7, 8, 9, 10).

Separate from DNA binding, but involving some of the same residues, a nuclear localization sequence comprised of positively charged arginines and lysines within the ETS facilitates nuclear transport via importin interactions (11, 12). Finally, specific ETS residues mediate protein–protein interactions with transcriptional partners, including c-Jun, C/EBPβ, GATA1, and RUNX1.

Unlike the well-described structure of the ETS domain, the acidic, GR, and PEST domains of SPI1 are intrinsically disordered (Fig. 1 B) (13). Intrinsically disordered regions (IDRs) are enriched in eukaryotic TFs and, despite functional importance, defy structural resolution using currently available technology (13, 14, 15). Although the ETS alone is sufficient to bind DNA at a 1:1 ratio, emerging data suggest the IDR, comprised of the acidic, GR, and PEST domains, is a critical interface for SPI1 homodimerization, representing a novel 2:1 structure-dependent regulatory mechanism (13). Within the IDR, the PEST domain contains serine residues, S^140^ and S^146^, which mediate heterotypic protein interactions once phosphorylated by casein kinase II or protein kinase C (16, 17, 18).

### SPI1 as a pioneer TF

Pioneer TFs, by definition, possess the unique ability of bypassing epigenetic regulation by binding to nucleosomal DNA of closed or hypermethylated chromatin to “pioneer” cellular reprogramming and direct lineage development and differentiation (15, 19). SPI1’s role as a hematopoietic pioneer TF has been extensively reported in both murine and human biological models (7, 20, 21, 22, 23, 24, 25). The effectiveness of SPI1 as a pioneer TF is predicated on four key molecular features. First, SPI1 resides in the nucleus in the basal state, as directed by importin α/β or Nup62/153 nuclear localization apparatuses (11, 12). This process is mediated by the ETS domain and, in the case of importin β, achieves this through robust collaborative interactions with positively charged residues at the C terminus (Fig. 1 B) (11). Second, SPI1 has specificity for methylated regions of the genome, thus bypassing a form of epigenetic regulation (21, 25). Third, SPI1 plays a key role in the Switch/Sucrose Non-Fermentable (SWI/SNF) chromatin remodeling complex (26). Here, the intrinsically disordered N-terminal portion of SPI1 (Fig. 1 B) interacts with SMARCA4/SMARCD1/SMARCC1, forming the canonical BRG/BRM-associated factor (BAF) complex to initiate chromatin remodeling (26). Fourth, direct interaction with SPI1 allows non-pioneer TFs to bind loci that would otherwise be inaccessible (22, 27). These non-pioneer TF partners are numerous and context dependent, requiring co-expression, and hence are the primary mechanism of pleiotropy in SPI1 biology.

### SPI1 transcriptional partners and cooperative/competitive interactions

**Figure 2.**
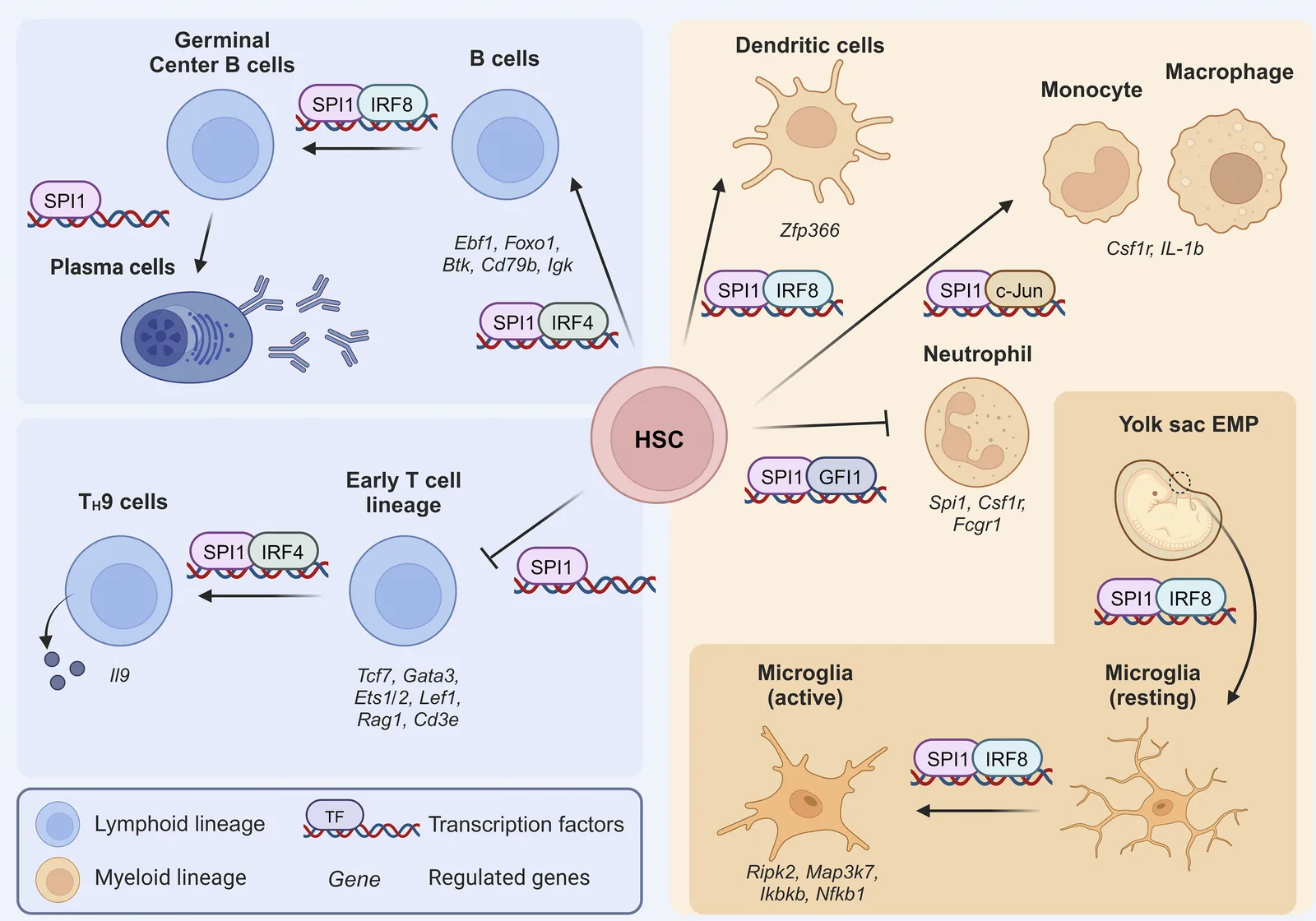
SPI1 co-regulates crucial steps during leukopoiesis beginning with HSCs. EMP, erythromyeloid precursor.

SPI1 can bind DNA directly as a monomer or homodimer but achieves more complex regulatory effects through cooperative interactions with partners (Fig. 2). Transcriptionally positive interactions with IRF4 or IRF8 in B cells and myeloid cells require phosphorylation of S^140^ and S^146^ residues in the SPI1 PEST domain (Fig. 1 B). Heterodimers of SPI1/IRF4 or SPI1/IRF8 bind to the purine-rich ETS-IRF composite element (EICE) GGAAnnGAAA. SPI1/IRF4/DNA ternary complex crystal structures indicate that DNA is contorted into an unusual S-shape that blocks stereotypic binding of IRF1 and IRF2 to the GAAA motif (16, 28, 29, 30, 31). A very different cooperative interaction occurs at the *Il1b* locus in murine macrophages. SPI1 uses R^230^ and R^233^ to tether the tail of the TF C/EBPβ bound to a distal enhancer located ∼3,000 base pairs upstream. The resulting long-range enhancer-to-promoter loop enables efficient and durable recruitment of transcriptional machinery (32, 33, 34, 35).

Although SPI1 lacks its own repressive domain, it can exert negative regulation in mice by partnering with HDAC1 and polycomb repressive complex 2 (PRC2) to silence genes via addition of H3K27me3 marks to their promoters (35, 36). SPI1 also discourages murine T cell lineage commitment in lymphoid progenitors by sequestering RUNX1 and SATB1, preventing them from positively regulating genes including *Rag1* (37).

### Transcriptional and post-translational regulation of *SPI1*/SPI1

*SPI1* is positively autoregulated through proximal and distal SPI1 to 5′-GGAA-3′ binding within its own cis-regulatory elements (CREs) (38). In mice, a distal CRE ∼14 kb upstream of *Spi1* is responsible for ∼80% of SPI1 expression across the hematopoietic compartment (39, 40). Additional CREs fine-tune SPI1 expression through binding of lineage-specific TFs such as C/EBPa, NF-κB, GATA-1, and GFI-1 (41, 42, 43, 44). SPI1 is also regulated by the noncoding RNA miR-155, which targets the 3′ untranslated region of *SPI1* mRNA to prevent protein translation (45, 46). miR-155 is induced by NF-κB and STAT5 pathways and is repressed by BCL6 (47).

After translation, several reported protein-to-protein interactions, both with itself and other proteins, regulate SPI1 abundance and function. SPI1 can be negatively regulated after it is translated through homodimerization. When in excess, SPI1 preferentially forms a homodimer via PEST/PEST interactions (13). Compared with monomeric SPI1, which binds DNA at a 1:1 ratio to drive transcription, SPI1 homodimers bind DNA at 2:1, which diminishes transactivation. SPI1 homodimerization provides a concentration-dependent negative feedback loop, especially vital for a TF that positively autoregulates the gene that encodes it. SPI1 can also be negatively regulated by ETS domain-mediated protein–protein interactions. GATA proteins (such as GATA-1) act in a mutually inhibitory capacity with SPI1 to specify cell lineage (48, 49) via an interaction that does not require SPI1 phosphorylation and cannot be perturbed by mutagenesis of single amino acids (49). It is proposed that GATA1 competes for binding with c-Jun, a coactivator of SPI1, with a weak interaction that is solely regulated by the ETS residues (49, 50, 51). The c-Jun–SPI1 heterodimer, however, is hinged at two nonredundant, highly conserved SPI1 β2 sheet ETS residues (Q^200^, F^201^; Fig. 1, B–D) and supports transcriptional processes in murine myeloid development (Fig. 2) (52). GFI-1 is another factor that directly interacts with the SPI1 protein and represses its activity (Fig. 2) (53). Testing of in vitro truncated alleles by co-immunoprecipitation revealed that this SPI1:GFI1 interaction is mediated by the four zinc fingers most proximal to the C terminus in GFI-1 and both an acidic/GR domain region (between aa33–100) as well as the ETS domain of SPI1 (53).

### SPI1 paralogs

SPI1 is a member of the ETS family of TFs, a 28-member family with considerable biological pleiotropy despite each having exquisite specificity for the 5′-GGAA-3′ DNA motif as directed by structurally homologous ETS domains (5, 9). The persistence of multiple and functionally diverse ETS family paralogs within complex mammalian species is likely indicative of a selective advantage (54, 55). Paralogs in other SPI TFs (SPIB, SPIC) have a highly conserved ETS sequence, particularly at key structural α helices and β sheets (Fig. 1 C). This likely explains similarity in the differentiation and function of mature B lymphocytes in murine models where either SPI1 or SPIB has been conditionally deleted (56, 57). ETS domain conservation is minimal between SPI1 and the two least phylogenetically related ETS TFs (ETS1, ETS2) and the only other ETS TF identified as a human monogenic inborn error of immunity (ELF4) (Fig. 1 C) (58). Encoded structural differences between the ETS family members have been shown to affect hydration at the ETS:DNA-binding interface, creating differential sensitivity to osmolarity between SPI1 and ETS1 (59, 60). However, there remain specific residues positioned across the ETS domains of the TFs that are completely conserved, especially within the α3 helical motif, indicating a degree of evolutionary intolerance to variation (Fig. 1 C). This fine-tuned conservation in some residues within ETS domains from this TF family likely contributes to their specificity for nearly identical DNA motifs (54).

## Cell type–specific roles

SPI1 is expressed across leukocyte lineages, where it acts either alone or with cell type–specific transcriptional partners to enforce a variety of critical gene expression programs. Key roles for SPI1 have been described in the following mouse and human immune cell types (Table 1).

**Table 1.** Phenotypic and functional consequences of genetic alterations to *SPI1* in mice or humans

Phenotype	Mice	Human
HSCs and multipotent progenitors	• Germline *Spi1*^−/−^ is embryonically lethal (67) • Germline *Spi1*^−/+^ mice have intact hematopoiesis (115, 131) • Reconstitution of bone marrow with *Spi1*^*−/−*^ HSCs resulted in reduced HSC number and loss of all common lymphoid and myeloid progenitors (115) • Compound knockout and knockdown of germline *Spi1* alleles (reducing SPI1 activity by 80%) causes rapidly developing AML (132)	• CITE-seq on a SPI1 haploinsufficiency patient had intact populations of HSCs and multipotent progenitors (22) • No patients with SPI1 haploinsufficiency have reported hematological malignancy (22, 70) • Somatic, hypomorphic SPI1 variants have been found in patients with AML (133, 134)
Pro- to pre-B cell transition	• Spi1 supports gene transcription in early B cell development at multiple non-redundant loci (e.g., *BTK*,* FOXO1*, *EBF*, and *CD79B*) (61, 75, 76, 135, 136, 137) • Conditional *Spi1*^−/−^ (*Cd19-*Cre) does not block B cell development (77, 115)	• SPI1 haploinsufficiency arrests B cell development at the pro- to pre-B transition (22)
Mature B cells	• Conditional *Spi1*^*−/−*^ (*Cd19-*Cre) does not affect class-switch recombination and Ab responses despite increased in vitro apoptosis following BCR cross-linking (77) • Crossing mice with conditional *Spi1*^*−/−*^ (*Cd19*-Cre*)* with *IRF8*^*−/−*^ results in reduced follicular B cells but an expanded population of plasmablasts (81)	• Mature B cells are absent from PU.MA patients (22, 70) • A SPI1 gain-of-function allele (p. Q226E) has been observed in up to 6% of tumor biopsies from patients with WM (138)
T cells	• *Spi1* downregulation is essential for pro-T lineage maturation at the DN2 to DN3 stage (87, 88) • Conditional *Spi1*^*−/−*^ at the DP T cell stage (*Lck*-Cre) develop to WT mice identically; however, they have an enhanced response to TCR stimulation and reduced maturation into IL-9–producing T_H_9 cells (86, 92)	• T cell development in PU.MA patients is largely intact (22, 70)
Neutrophils	• *Spi1*^*−/−*^ mice have incomplete neutrophil development (granulocytic morphology, lack CD11b expression) and perturbed chemotaxis due to poor response to chemokines, and inferior bacterial clearance due to poor bacterial uptake and superoxide ion production (113) • Normal neutrophil phenotype in neutrophil-specific *Spi1*^*−/−*^ (*Mrp8*–Cre); however, these neutrophils were hyperinflammatory with increased degranulation and enhanced migration into Candida-infected tissues, despite impaired ROS production (116)	• PU.MA patients are not neutropenic (22, 70)
Monocytes and macrophages	• Germline *Spi1*^*−/−*^ mice have undetectable monocytes and macrophages in fetal liver tissue (118) • Conditional *Spi1*^*−/−*^ (*Mx*-Cre) with poly I:C ablates development of monocytes and macrophages, skewing myelopoiesis towards the granulocyte fate (67) • Tamoxifen-induced *Spi1*^*+/−*^ mice have a partial decrease in myeloid development (by CD11b expression) in fetal liver cells; bone marrow–derived macrophages (BMDMs) have reduced inflammatory markers (COX-2, iNOS, IL-6, and TNF) in response to LPS (121) • Bone marrow chimera models depict a graded reduction (*Spi1*^*−/+*^ > *Spi1*^*−/−*^) in cell-intrinsic SPI1–NF-κB-mediated BMDM inflammation in response to LPS (121)	• PU.MA patients have circulating monocyte subsets (22)
DCs	• Conditional *Spi1*^*−/−*^ with poly I:C in *Mx*-Cre mice reduces development of both cDCs and pDCs in a cell-intrinsic manner (62) • Conditional *Spi1*^*−/−*^ in committed DCs (*Itgax*-Cre) results in decreased cDCs but enhanced pDCs (122)	• Reduced cDC and pDC maturation in PU.MA patients (22, 70)
Microglia	• Germline *Spi1*^*−/−*^ mice have ablated development of both A1- and A2-myeloid progenitors which seed the microglial cell pool (124) • Tamoxifen-induced conditional *Spi1*^*−/−*^ in microglia (*Cx3cr1*-Cre) blunts activation, proliferation, and morphology following optic nerve injury (125)	• Multiple Alzheimer’s risk alleles in *SPI1* reduce *SPI1* expression and are speculated to perturb gene regulatory networks for microglia development and function (139)

### Hematopoietic stem cells (HSCs) and multipotent progenitors

SPI1 is expressed in HSCs and multipotent progenitors. At initial stages in mouse leukopoiesis, monomeric SPI1 positively regulates *IL7RA* and *FLT3* to drive progenitor proliferation and survival, respectively (61, 62). Complete SPI1 deficiency is embryonically lethal in mice due to total loss of lymphoid and myeloid lineages (63). Complete SPI1-deficient humans have not yet been described, and the lack of biallelic loss-of-function SPI1 variants in large, publicly available genomic databases like gnomAD (64, *Preprint*) suggests that leukopoiesis (and postfetal life) is SPI1 dependent across mammalian species.

Differential SPI1 expression among hematopoietic progenitors has been proposed to determine myeloid and B cell lineage fate (65). This model is based on renowned experiments performed by Dekoter and Singh that restored leukopoiesis in complete SPI1-deficient murine progenitor cells with a SPI1-producing retrovirus. Since the macrophages derived from these experiments expressed higher SPI1 than counterpart B cells, these authors concluded that low retroviral transduction (i.e., low SPI1 dose) favored B cell lineage commitment, whereas high SPI1 expression specified myeloid lineage fate. The model has been challenged by other groups who found uniform SPI1 expression across adult mouse HSCs, common lymphoid progenitors, and common myeloid progenitors (CMPs) (66, 67, 68). In SPI1-sufficient mice and humans, circulating myeloid cells (e.g., monocytes, dendritic cells [DCs]) do express much more SPI1 than B cells, but this may be a consequence, not a cause of, lineage choice. Indeed, genetically engineered mice that express 80% lower levels of SPI1 by deletion of a start codon leading to alternative start codon usage exhibit blocked early B cell development and induced myeloproliferation (69). This animal model partially mirrors human SPI1 haploinsufficiency, where early B cell development is completely blocked at the pro- to pre-B cell transition, while most myeloid subsets are numerically preserved (22, 70). So, murine and human leukopoiesis are certainly altered by lower SPI1 doses, but it is B cells, not myeloid cells, that are most negatively affected by the decrease.

### Early B cell development

On their way to becoming mature, B cell progenitors must carefully sequence multiple priorities, including lineage commitment, clonal proliferation, heavy chain recombination, pre-B cell receptor (BCR) signaling, more clonal proliferation, light chain recombination, BCR expression, and central immune tolerance (71, 72). A suite of TFs, including SPI1, IKZF1, E2A, ETS1, EBF1, PAX5, FOXO1, IRF4, IRF8, and IKZF3, coordinate the effort (73, 74). During the pro-B cell stage, monomeric SPI1 works cooperatively with other TFs to induce EBF1, which enforces B-lineage commitment; FOXO1, which allows RAG to generate an IGH heavy chain; and several components of the pre-BCR signaling complex, including BTK and CD79β (22, 75). At the pre-BCR stage in mice, IRF4 becomes abundant, and SPI1/IRF4 heterodimers cooperatively bind EICEs at the 3′ κ and λ enhancers, facilitating BCR light chain recombination (Fig. 2) (76).

### Mature B cell development

After the pro-B cell stage, SPI1 plays a less determinative role. Lineage-specific complete SPI1 deletion with *CD19*-Cre recombinase does not block murine B cell differentiation (class-switch recombination and immunoglobulin secretion) but does subtly alter B cell survival in vitro following BCR cross-linking (77, 78). SPI1 dispensability in mature B cells has been posited to be due to redundancy between SPI1 and its paralogue SPIB. While SPI1 is expressed early in HSCs, SPIB is first upregulated in pro-B cells. SPI1 and SPIB possess a high degree of amino acid homology in the DNA-binding domain (Fig. 1 C), occupy similar target sites, and exhibit overlapping gene regulation (79, 80). Sokalski and colleagues tested B cell lineage dependency on SPI1 and SPIB at lineage commitment using *Cd19*-Cre recombinase SPI1^lox/lox^ and SPIB^lox/lox^ mice. Loss of both TFs reduced circulating B cell frequencies more than loss of either alone (56). Willis and colleagues investigated SPI1/SPIB redundancy at later B cell stages using *Cd23*-Cre mice that generate mature B cells that are SPI1 deficient, SPIB deficient, or both (57). Loss of either TF alone had few appreciable consequences, whereas combined loss conferred defects in germinal center (GC) formation, antigen-specific responses, and GC B cell survival (57). SPI1/IRF8 heterodimerizes to maintain BCL6 in GC B cells and antagonize BLIMP1 expression (Fig. 2) (81, 82). Rising IRF4 and falling SPI1 concentrations during plasmablast differentiation permit IRF4 homodimerization, which antagonizes BCL6, promotes BLIMP1 expression, and favors terminal plasma cell differentiation (Fig. 2) (83, 84).

### T cells

SPI1 is required to generate all lymphocytes before T cell lineage commitment (85). However, complete deletion of *Spi1* in the developing murine T cells (double-positive [DP] stage, *Lck-*Cre) has little effect on mature T cells besides an enhanced response to TCR stimulation (86). Instead, experiments in mice where *Spi1* was constitutively expressed by retroviral vectors in hematopoietic precursors through differentiation indicate that it must be downregulated during the pro-T cell stage to support T lymphocyte lineage maturation (Fig. 2) (87, 88). Later and in special contexts, SPI1 can be re-expressed during T_H_9 cell development (89, 90, 91, 92, 93). T_H_9 cells co-opt portions of T_H_2 and T_H_17 differentiation pathways, including common developmental cues (IL-4 and TGFβ) and TFs (GATA3, IRF4, BATF, and PPAR-γ) (89, 90, 94, 95). Once developed, Th9 cells are a key source of IL-9 but also produce T_H_2 cytokines IL-4, IL-10, and IL-13. IL-9 is also secreted in significant quantities by T_H_17 cells (96).

IL-9 mediates anti-parasitic, antifungal, and antiviral immune responses at barrier sites (lung, skin, and gut) through eosinophil and mast cell recruitment, increased mucus production, and B cell class-switching to IgE (97, 98, 99, 100, 101, 102, 103, 104). An EICE sequence within the *IL9* gene promoter is bound by a SPI1/IRF4 heterodimer, and the loss of either TF compromises T_H_9 cell differentiation in mice (Fig. 2) (92, 105). Other animal models demonstrate a complex control architecture for *Il9* involving a suite of TFs, including ERG, ETV5, IRF1, BATF, NFAT, SMAD2/3/4, STAT5/6, GATA3, and NF-κB (106, 107, 108). Among human inborn errors of immunity patients, in vitro differentiation of IL-9–producing CD4^+^ T cells is abrogated by loss of IRF4, STAT3, JAK1, and STAT5B and augmented by constitutively increased STAT6 signaling. These results reinforce the need for cooperative action by signaling molecules and TF networks downstream of cytokine receptors for IL-9 expression in humans. The effects of human SPI1 haploinsufficiency on T_H_9 cell differentiation have not yet been formally investigated.

### Granulocyte-monocyte progenitors

At the granulocyte-monocyte progenitor (GMP) stage, SPI1 is a pivotal regulator of monocyte versus neutrophil fate choice in mice. Foundational loss- and gain-of-expression murine studies support a dosage-dependent model in which lower SPI1 activity supports granulocytic differentiation, whereas higher SPI1 expression promotes monocytic differentiation by inducing expression of TFs that suppress granulocyte development; this balance is further shaped by interactions with other lineage-defining regulators, including GFI-1, which supports neutrophil specification, and IRF8, which promotes mononuclear phagocyte differentiation while restraining neutrophil differentiation (Fig. 2) (109, 110). By contrast, C/EBPα acts earlier and more broadly in myeloid specification, with context-dependent effects that vary according to SPI1 induction and cooperating transcriptional partners (110, 111). This classical framework has been substantially refined by recent single-cell studies in mice and humans showing that phenotypically defined GMPs comprise a heterogeneous mixture of progenitors rather than a uniform bipotent intermediate (112). Notably, these include progenitors aligned with both monocyte and neutrophil trajectories, such as common monocyte progenitors and the early unipotent neutrophil progenitor proNeu1 (112). However, it remains unclear how SPI1 dosage, timing, and TF cooperativity operate across these distinct progenitor states to direct lineage commitment in vivo.

### Neutrophils

SPI1 has long been recognized as an important regulator of neutrophil terminal maturation and antimicrobial competence in mice. During granulocyte differentiation, GFI-1 physically interacts with SPI1 and represses SPI1-dependent transcription, preferentially suppressing macrophage-associated target genes such as *Csf1r* and *Fcgr1*, which encode M-CSFR and CD64, respectively. By limiting inappropriate macrophage gene expression, this antagonism is thought to stabilize granulocyte identity rather than directly initiate granulocyte commitment (53). In conventional SPI1-null settings, murine neutrophil-like cells acquire granulocyte-like segmented morphology, Gr-1 expression, and primary granule gene expression, yet they fail to express CD11b, lack secondary granule gene expression, and show defective chemotaxis, respiratory burst, phagocytosis, and bacterial killing, indicating incomplete terminal maturation and impaired functional competence (113, 114). Consistent with this view, a conditional deletion study showed that SPI1 ablation at the CMP/GMP stage (*Mx1*-Cre mice receiving polyinosintol:polycytidylic acid (poly I:C) injection 1–2 days after birth) did not prevent colony formation but blocked maturation into CD11b^+^ myeloid cells, instead yielding immature myeloblast colonies, supporting a continued requirement for SPI1 after myeloid commitment (115). However, neutrophil-selective deletion of *Spi1* in the MRP8–Cre system did not reproduce the loss of CD11b expression or the pronounced accumulation of immature neutrophil precursors described in earlier *Spi1*-loss models. CD11b and Ly6G expression remained normal, and although c-Kit downregulation was delayed in the bone marrow, substantial numbers of neutrophils lost c-Kit expression, acquired mature multilobulated nuclear morphology, and populated the periphery, indicating that terminal maturation was not completely blocked (116). The predominant abnormalities instead involved neutrophil effector functions, with *Spi1*-deficient neutrophils exhibiting intrinsic hyperactivity, enhanced tissue-destructive potential, accompanied by increased degranulation, and enhanced migration into *Candida*-infected tissues, despite impaired ROS production (116). Why overt maturation defects were not observed, despite the established requirement for SPI1 in earlier stages of neutrophil development, remains incompletely understood. These observations support a role for *Spi1* in neutrophil maturation in mice, while highlighting unresolved stage- and model-dependent differences in the phenotypic consequences of SPI1 loss. However, studies directly examining the effects of germline SPI1 mutations on neutrophil maturation and effector functions in humans have not yet been reported. Further studies are required to define the precise developmental window during which SPI1 is required for neutrophil differentiation, clarify its stage-specific functions, and establish relevance of effects on neutrophils to human disease.

Beyond its roles in canonical neutrophil maturation and effector function, SPI1 may also regulate neutrophil plasticity. One study showed that FcγR engagement can drive mature murine neutrophils toward a CD11c^+^MHCII^+^ APC-like state, and both pharmacologic inhibition of SPI1 and neutrophil-specific SPI1 deletion impaired this conversion and reduced the ability of these cells to stimulate CD4^+^ and CD8^+^ T cells (117). Notably, similar findings were observed in human neutrophils: engagement of FcγRIIIB promoted conversion into CD11c^+^HLA-DR^+^ APC-like cells, and this process was abrogated by pharmacologic SPI1 inhibition, supporting a conserved requirement for SPI1 activity in stimulus-induced neutrophil reprogramming (117). These findings suggest that SPI1 is required not only for neutrophil differentiation but also for stimulus-induced transcriptional reprogramming into alternative functional states.

### Monocytes and macrophages

Compared with granulopoiesis, monocyte-to-macrophage differentiation appears to depend on a more robust and sustained SPI1-dependent transcriptional program. Early mouse studies showed that SPI1-deficient progenitors failed to generate monocytes/macrophages even under myeloid cytokine conditions that still permitted granulocytic development, indicating a selective cell-intrinsic requirement for SPI1 in macrophage lineage specification (118). At the GMP stage, high SPI1 dosage contributes to lineage bias by inducing macrophage-associated regulators and repressing neutrophil-promoting programs, thereby favoring macrophage over granulocyte fate (112, 119). Beyond lineage choice, SPI1 promotes macrophage differentiation and identity by activating myeloid receptor programs and maintaining the accessibility of macrophage-specific gene loci. PRC2 mediates transcriptional silencing through trimethylation of H3K27me3. In a macrophage differentiation model, SPI1 binding at regulatory elements prevented PRC2 recruitment and maintained low nucleosome occupancy, preserving the capacity of associated genes to respond to subsequent stimulation. In the absence of SPI1 binding, these loci accumulated PRC2 and H3K27me3, became less accessible, and failed to undergo stimulus-induced transcription (120).

SPI1 also cooperates directly with c-Jun, an AP-1 family member, to promote monocyte/macrophage differentiation in mice (Fig. 2) (52). c-Jun can physically interact with SPI1 and enhance SPI1-dependent transcriptional activation, including at myeloid genes such as *Csf1r*, and disruption of the SPI1–c-Jun interaction impairs monocyte/macrophage differentiation (52). In mature macrophages, SPI1 further contributes to inflammatory activation, including LPS-induced NF-κB responses, and may support alternative activation in a context-dependent manner (121). Together, these findings place SPI1 not only at the point of monocyte commitment but also in the establishment and maintenance of macrophage identity, while its exact contribution to distinct tissue-resident and human macrophage states remains incompletely defined.

### DCs

Within the DC lineage, SPI1 helps establish both developmental competence and functional identity. In mice, SPI1 is required not only for DC development but also for DC subset specification and mature function (62, 122). SPI1 supports DC development in a dose-dependent manner, in part through regulation of FLT3, and reduced SPI1 preferentially impairs conventional DC (cDC) output (62). More specifically, conditional deletion of *Spi1* in DC-restricted progenitors increased plasmacytoid DC (pDC) formation at the expense of cDCs, indicating that sustained SPI1 expression is required to promote cDC identity while restraining pDC differentiation (122). Mechanistically, SPI1-deficient cDCs derepressed the pDC transcriptional program and lost expression of multiple cDC-associated genes, while SPI1 directly activated the DC-specific transcript (DC-SCRIPT, encoded by *Zfp366*), which in turn promoted cDC differentiation, particularly along the cDC1 pathway (Fig. 2) (122). SPI1 also remained functionally important in mature murine DCs, where its loss impaired antigen presentation, reduced induction of co-stimulatory molecules, decreased cDC survival, and blunted IFNα production by pDCs (122).

**Figure 3.**
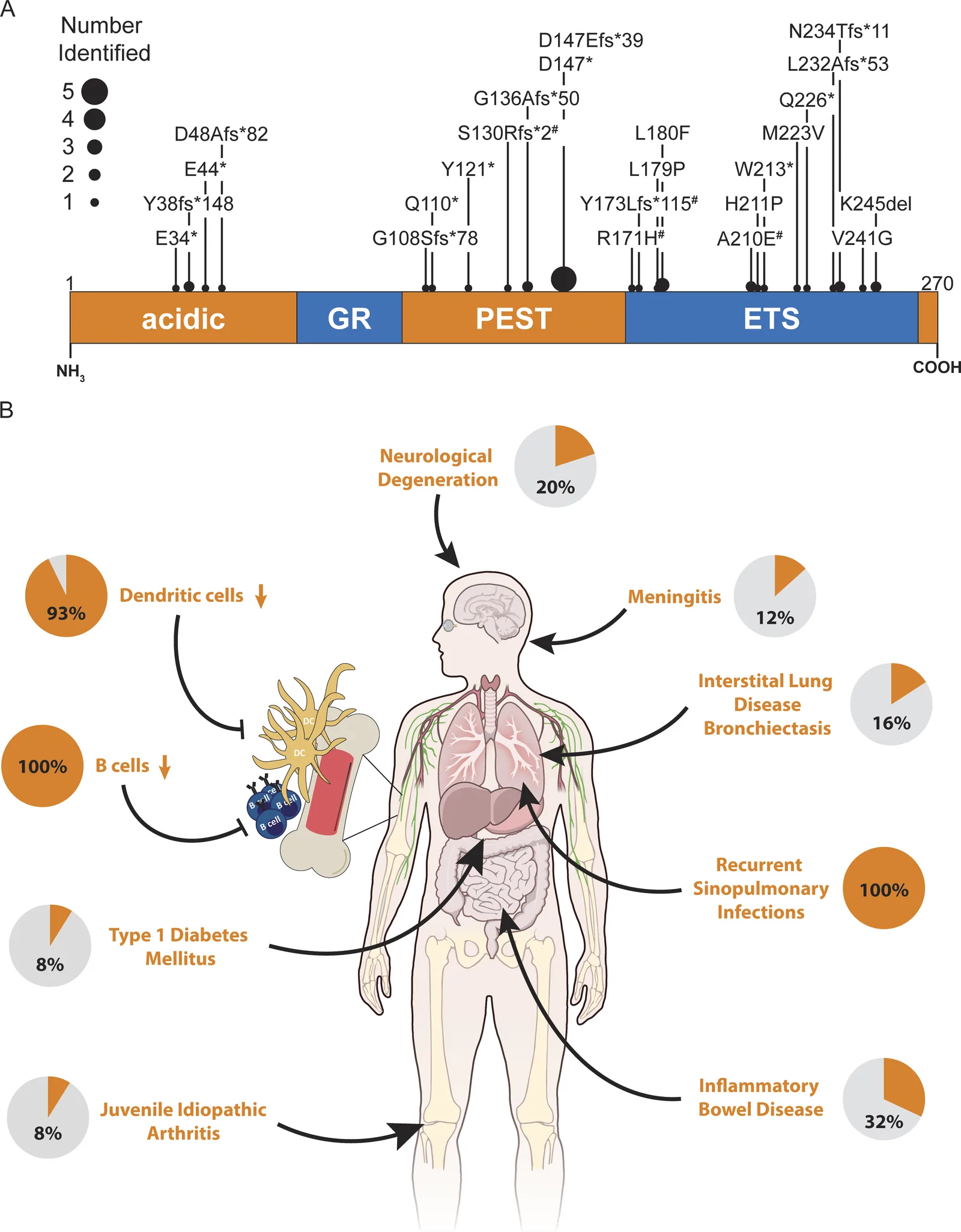
**Mutational and clinical landscape of PU.MA. (A)** Location of functionally validated heterozygous loss-of-function *SPI1* variants from affected PU.MA patients. Unpublished loss-of-function variants are denoted “#.” The number of patients identified with each loss-of-function variant is displayed. **(B)** Frequency of clinical and laboratory manifestations experienced by PU.MA patients (23 previously published, 3 unpublished) are represented.

Direct human evidence is more limited. The strongest human data come from monocyte-derived DCs, where DC-SCRIPT regulates IL-12 expression and T cell responses, and from studies linking SPI1 to MHC class II regulation (122, 123). Although circulating cDC and pDC frequencies are both lower in SPI1 agammaglobulinemia patients (Fig. 3 B), cDCs are especially scarce (70), suggesting SPI1 influences cDC-versus-pDC lineage balance in human DC progenitors.

### Microglia

SPI1 is indispensable for microglial ontogeny and remains functionally relevant after development (124, 125). Foundational studies in mouse embryos established that microglia arise from yolk sac–derived c-Kit^+^ erythromyeloid precursors that progress through CX3CR1^−^ and then CX3CR1^+^ immature intermediates before colonizing the embryonic brain, and that this developmental sequence requires SPI1 together with IRF8 (Fig. 2) (124). Mechanistically, SPI1 deficiency reduces early A1 and A2 progenitor populations, whereas IRF8 deficiency preferentially impairs maturation of the A2 population, indicating that these factors operate at distinct but linked stages of microgliogenesis (124). SPI1 also remains important after development, as postdevelopmental deletion of SPI1 in microglia under neurodegenerative conditions suppresses activation-associated transcriptional programs and blunts proliferative and morphological responses (125). In this context, SPI1 cooperates through composite IRF-ETS motifs, and the two factors positively reinforce each other’s expression, forming a transcriptional module that sustains the activated microglial state (125). Evidence from human systems, although still more limited than in mice, supports a conserved role for SPI1 in microglial identity and immune function (126, 127). In primary human microglia, SPI1 knockdown altered gene networks related to antigen presentation and phagocytosis and reduced expression of mediators such as DAP12 and HLA-DR/DP/DQ, identifying SPI1 as a highly connected regulator of innate and adaptive immune gene programs (126). Complementary organoid-based studies showed that enforced SPI1 expression can generate microglia-like cells within human cortical organoids, yielding cells with phagocytic activity, complement and chemokine signatures, and partial transcriptional similarity to fetal human microglia (127). Together, these findings support a model in which SPI1 helps establish microglial lineage identity during development and later regulates activation and disease-relevant response programs. The extent to which developmental mechanisms defined in mice map directly onto human microglia remains unresolved.

## *SPI1* genetic alterations in human diseases

### Inborn PU.1-mutated agammaglobulinemia (PU.MA)

Heterozygous loss-of-function of SPI1 is a monogenic cause of agammaglobulinemia, termed PU.MA. Affected patients lack circulating B cells and antibodies (Fig. 3, A and B) (22, 70). SPI1 haploinsufficiency confers a developmental block at the pro-B to pre-B cell transition, a stage when demand for SPI1 and SPI1 target genes such as *BTK* are highest. SPI1 haploinsufficiency also diminishes circulating cDC and pDC frequencies (Fig. 3 B). At least 26 pathologic SPI1 variants have been identified in agammaglobulinemic patients, most arising de novo, although familial cases have been described (Fig. 3 A) (22, 70, 128, 129, 130). Molecular mechanisms leading to SPI1 loss of function include *SPI1* transcript instability, SPI1 protein loss, blocked nuclear localization, and inability to bind chromosomal DNA. Genetic variants encoding dominant interfering SPI1 proteins have not yet been described.

The effects of SPI1 haploinsufficiency in humans appear incompletely penetrant with variable expressivity. Human subjects with heterozygous loss-of-function SPI1 variants have been identified with circulating B cells; some have mild antibody deficiency, and others appear overtly healthy. Among those affected with agammaglobulinemia, disease presentation is most similar to BTK-deficient X-linked agammaglobulinemia (XLA) patients. PU.MA patients typically experience recurrent bacterial sinopulmonary infections (100%), commonly resulting in structural lung damage (interstitial lung disease or bronchiectasis, 16%) and either bacterial or enteroviral central nervous system (CNS) infections (12%, Fig. 3 B). Disease onset occurs most often in the first year of life, although initial presentation in older children and young adults has been reported (70, 129). Noninfectious disease sequelae include inflammatory bowel disease (∼32%), neurocognitive disorders (∼20%), juvenile idiopathic arthritis (8%), and type 1 diabetes (8%, Fig. 3 B) (70, 130). The affected patients reported to date have not experienced hematologic malignancies.

The current mainstay of treatment for SPI1 agammaglobulinemia is lifelong immunoglobulin replacement therapy (IRT). HSC transplantation (HSCT) has been attempted in three SPI1 agammaglobulinemia patients. For unclear reasons, transplant outcomes have been poor: one patient rejected their graft and developed late catastrophic neurologic deterioration, another remains chronically dependent on IRT, and the last reached IRT independence in the first year but suffered transplant-related paralysis from viral encephalitis (70, 128).

### Somatic SPI1 alternations in Waldenstrom’s macroglobulinemia (WM)

WM is a lymphoplasmacytic lymphoma with a monoclonal spike of a pentameric IgM protein (140). In WM, malignant B cell development arrests at the transition between postgerminal center IgM^+^ memory B cells and plasma cells. Serum IgM concentration in WM is often increased 50- to 100-fold, causing a dangerous hyperviscosity syndrome, peripheral neuropathy, hemolytic anemia, and immune complex vasculitis. Various somatic activating gene mutations are associated with WM, including in *MYD88* (95% of cases), CXCR4 (28%), and *SPI1* (6%) (138, 141, 142). The recurrent WM-associated SPI1 mutation, Q226E, localizes to the ETS domain. SPI1 Q226E retains binding to the classic SPI1-binding motif and demonstrates two- to threefold increased transactivation of some but not all target genes. SPI1 Q226E also exhibits neomorphic affinity and new transactivation capacity at ETS1-like binding sites. Its biologic consequences in B cells include blocked terminal maturation and increased cellular proliferation via constitutively active MYC, BCR, CD40, TLR, and PI3K signaling. Although overall survival is significantly reduced for WM patients carrying somatic SPI1 Q226E mutations, promising in vitro data suggest JQ1, a BET inhibitor that targets MYC, and lenalidomide, which targets the SP1/IRF4 heterodimer, could be effective personalized therapies (138).

### Somatic SPI1 alterations in acute myeloid leukemia (AML)

Mouse studies established that SPI1 dosage is a critical determinant of myeloid leukemogenesis: an ∼80% reduction in SPI1 expression induces AML, whereas simple haploinsufficiency does not (132). More modest SPI1 reduction can still be pathogenic in the appropriate genetic context, as a ∼35% decrease was sufficient to generate myeloid-biased preleukemic stem cells and promote progression to AML in mismatch repair-deficient mice (143).

In human AML, coding mutations of *SPI1* appear to be uncommon overall, despite early reports describing heterozygous mutations in a subset of cases (133, 134). Subsequent studies instead suggest that human AML more often involves quantitative or regulatory impairment of SPI1 than biallelic coding disruption (143, 144). Consistent with this view, reduced SPI1 expression has been described in AML subtypes such as FLT3-ITD, RUNX1-ETO, and acute promyelocytic leukemia, and lower SPI1 levels are also found in a substantial fraction of stem cells purified from AML patients (144). Reduced SPI1 activity may support leukemic cell survival through compensatory transcriptional rewiring, including RUNX1-associated activation of survival and autophagy-linked genes (145). Counterintuitively, small-molecule inhibitors that allosterically interfere with SPI1 chromatin binding have shown therapeutic promise when applied to murine and human AML cells in vitro (144, 146).

In *MLL*-rearranged AML, SPI1 is required for leukemic stem cell maintenance by supporting expression of the M-CSF receptor CSF-1R; more generally, pharmacologic or genetic SPI1 inhibition suppresses murine and human AML by promoting myeloid differentiation (144, 147, 148). Thus, current evidence supports a context-dependent model in which reduced SPI1 dosage can contribute to AML initiation, whereas residual SPI1 activity remains necessary for maintenance of established leukemia in selected molecular settings (143, 144, 147, 148). Notably, this association has not clearly extended to germline SPI1 haploinsufficiency, as no hematologic malignancies have been reported among the 25 SPI1 agammaglobulinemia patients and 11 heterozygous relatives described to date (22, 70).

## Potential contributions of SPI1 to immune-mediated and CNS diseases

### Rheumatoid arthritis (RA)

Evidence linking SPI1 to RA is growing, but its effects appear strongly cell type and context dependent. In human RA synovium, SPI1 is increased relative to osteoarthritis or normal synovium and is detected in macrophages, fibroblast-like synoviocytes (FLS), CD4^+^ T cells, and B cells (45, 149, 150, 151, 152, 153). Among these, the best-characterized pathway involves B cells and the miR-155 axis: RA B cells, particularly in anti-citrullinated protein antibody (ACPA)–positive disease and patients with synovial lymphoid follicles, show increased miR-155 together with reduced SPI1; miR-155 inhibition restores SPI1 and reduces antibody production by RA peripheral blood B cells (45).

By contrast, several studies support a pro-inflammatory role for SPI1 in macrophages and stromal cells (149, 151, 152). In RA macrophages, SPI1 promotes TLR4/MyD88/NF-κB signaling, whereas cilostazol reduces SPI1 recruitment to the nucleus and reduces cytokine production (149). More recent work further showed that SPI1 represses FLT3 and promotes inflammatory activation of macrophages and FLS, while SPI1 reduction or pharmacologic inhibition alleviates experimental arthritis and suppresses RA-FLS proliferation and cytokine expression (152).

SPI1 has also been linked to RA through T_H_9 biology (150, 153). IL-9, IL-9R, and SPI1 are increased in RA synovium, peripheral T_H_9 cells expand in response to citrullinated peptides, and recent data suggest that SPI1 and IL-9 form a positive feedback loop that amplifies T_H_9-driven inflammation (153). Together, these findings support SPI1 as a relevant but context-specific regulator in RA, with the major unresolved question being why SPI1 appears suppressive in some miR-155–associated settings yet pathogenic in macrophage/FLS- and T_H_9-centered models.

ACPA-positive RA has not been reported among the 25 SPI1 agammaglobulinemia patients described to date, which should not be surprising, as they uniformly lack the ability to generate autoantibodies. Notably, two patients developed juvenile idiopathic arthritis and one developed reactive arthritis in the setting of prolonged echoviremia (70).

### Systemic lupus erythematosus (SLE)

Similar to RA, the role of SPI1 in SLE appears to be cell type and context dependent, but overall available evidence points toward disease-promoting effects of increased or dysregulated SPI1 activity (154, 155). Genetic data first implicated *SPI1* in SLE susceptibility, as a functional 3′-UTR polymorphism (rs1057233) was associated with increased *SPI1* mRNA expression in lymphoblastoid cells and with SLE (odds ratio: 1.24 [1.02–1.51]), particularly nephropathy (odds ratio: 1.34 for SLE with nephropathy [1.05–1.73]) (156).

At the cellular level, SPI1 expression is increased in several immune compartments relevant to lupus. In SLE, SPI1 is elevated in CD4^+^ T cells, and its expression in these cells correlated with IL-1β, supporting a link to inflammatory cytokine programs (154). SPI1 has also been implicated in IL-9–associated T cell responses, supporting a role in cytokine-driven T cell inflammation (157). In pediatric SLE, SPI1 was also upregulated in peripheral blood mononuclear cells and B cells and has been positioned upstream of a SPI1/TNF-α/BAFF/CD19 axis that may enhance B cell activation and autoimmunity (158).

More recently, SPI1 has been linked to innate immune dysregulation in lupus through impaired C1q control (155). In humans with SLE and in murine lupus models, posttranslational modification of SPI1 by isolevuglandin adduction reduced its binding to the shared C1q promoter, lowered C1q expression, and promoted DC activation, autoimmunity, renal injury, and hypertension (155). Although SLE has not been reported in SPI1 agammaglobulinemia patients, who lack autoantibody-generating machinery, altered SPI1 expression among patients with B cells may contribute to SLE pathophysiology across diverse cell types.

### Atopic diseases and psoriasis

T_H_9 responses were first linked to human atopic pathology in patients with allergic asthma following genetic linkage analysis pinpointing the *IL9* locus to this disease in several human cohorts (159, 160). Bronchial biopsy from asthmatic patients had dramatic elevation in *IL9* mRNA transcript and IL-9 protein compared with healthy donors, while bronchial biopsy from non-asthmatic atopic patients had only mild increases when compared with healthy donors (160). Targeting IL-9 with the humanized anti–IL-9 monoclonal antibody enokizumab has demonstrated mixed efficacy in treating patients with asthma (161, 162, 163).

The overlapping etiology of the T_H_9 and T_H_17 differentiation stages has birthed speculation of a potential role of SPI1 in psoriasis pathogenesis, considering the long-standing paradigm in which hyper-T_H_17 responses drive this disease. Elevated IL-9 has routinely been observed in the serum and skin lesions of psoriatic patient cohorts (94, 104, 164). It remains to be determined whether IL-9 is primary to pathogenesis or secondary to a localized inflammatory milieu in these cases. If germline, somatic hypermorphic or neomorphic *SPI1* variants are identified in psoriasis patients, this may provide future opportunities for disease-specific investigations.

### Inflammatory bowel disease (IBD)

Like RA and SLE, IBD appears to act across multiple immune compartments (165). Although studies mechanistically linking SPI1 to IBD remain limited, there is a strong literature of IgA deficiency-associated inflammatory bowel disease, as is seen in XLA (166) and common variable immune deficiency patients (167, 168). Another potential mechanism has been observed in murine T cell–mediated ulcerative colitis, where SPI1-dependent T_H_9 cells promote IL-9–dependent epithelial dysfunction and impaired mucosal healing (169). By contrast, human genetic studies have suggested that neutrophils may represent an important SPI1-linked effector compartment for selected IBD risk loci (170). Notably, gastrointestinal inflammatory disease is observed in SPI1 agammaglobulinemia, with 8 of 23 patients developing heterogeneous gastrointestinal inflammatory manifestations, including enterocolitis, sprue-like disease, perirectal abscess, and autoimmune gastritis (70).

### CNS disease

SPI1 has emerged as an important regulator of CNS disease, likely through its control of microglial identity and state transitions (126, 127, 171). In Alzheimer’s disease (AD), human genetic studies initially linked a SLE-associated 3′-UTR polymorphism (rs1057233) with lower *SPI1* expression in myeloid cells and delayed disease onset (odds ratio: 0.93 [0.89–0.96]) (172). Consistent with this interpretation, in vitro studies in microglia found that reduced SPI1 can dampen inflammatory and phagocytic programs (126, 139). However, in vivo findings have not been uniform. In one study, heterozygous *Spi1* knockdown in a murine APP/PS1 amyloidosis model increased Aβ aggregation, plaque burden, and gliosis, whereas *Spi1* overexpression in 5xFAD mice reduced amyloid pathology and glial activation (173). By contrast, more recent work using postnatally induced microglia-specific SPI1 reduction in 5xFAD mice identified a plaque-associated SPI1^low^ microglial state linked to reduced neuroinflammatory programs and protection from amyloid pathology (171). These studies suggest that the consequences of SPI1 reduction in AD cannot yet be generalized and likely depend on which cells are affected, when SPI1 is altered, and which model is used.

Outside AD, reduced SPI1 activity has been implicated in microglial dysfunction in human C9ORF72-associated amyotrophic lateral sclerosis/frontotemporal dementia, where a downregulated SPI1 regulon was associated with impaired phagocytosis and lysosomal defects, and SPI1 re-expression rescued these abnormalities (174). This broader CNS relevance may also extend to SPI1 agammaglobulinemia, in which neurocognitive disorders have been reported in 6 of 25 patients (70), including dramatically rapid and ultimately fatal neurodegeneration in one affected patient, iatrogenic poliomyelitis in a second, and posttransplant paralysis in a third (130). It is unclear what roles SPI1-dependent infectious susceptibilities versus microglial dysfunction played in each event.

## Conclusions

40 years after its discovery, key roles for SPI1 have been identified across leukopoiesis. Given SPI1’s ability to open heterochromatin and regulate the genes within, either by itself or with an array of transcriptional partners, it is not surprising that germline and somatic SPI1 alterations cause cascading transcriptional changes across the genome affecting multiple cell types. Although several SPI1-associated diseases have been described, no drugs directly targeting SPI1 are currently available as treatments. In vitro data using engineered small-molecule inhibitors and repurposed chemotherapy agents to treat SPI1-associated blood cancers are encouraging and theoretically could be repurposed for other indications. HSCT outcomes for SPI1 agammaglobulinemia patients have been disappointing so far, but restoration of SPI1 using gene editing is technically possible and may yield better results. Furthermore, cutting-edge in vivo gene-editing strategies now only available in research protocols (175) may be capable of restoring SPI1 in noncirculating immune cells such as microglia to address neurodegenerative pathologies.

Despite major advances, several conceptual and technical limitations constrain the current framework of SPI1 biology. A central challenge is model dependence: phenotypes differ substantially across constitutive, inducible, and lineage-restricted systems, complicating efforts to define a single hierarchy of SPI1 function. Further, SPI1 appears to exert distinct effects depending on expression level and developmental stage. Moreover, SPI1 function is highly context dependent, shaped by local tissue signals and cooperation with partner TFs, so conclusions drawn in one lineage or activation state may not generalize to others. An additional limitation is that much of the mechanistic evidence still comes from mouse models, whereas direct human validation remains uneven across cell types. Moving forward, the field will benefit from single-cell and multi-omic analyses, temporally precise perturbation strategies, and human systems that better capture endogenous development and disease context, allowing a more unified and testable understanding of how SPI1 regulates lineage specification, cell-state transitions, and immune effector function.
